# Revisiting intervertebral disc degeneration: lipid metabolism disorders as overlooked contributors to complex pathology

**DOI:** 10.3389/fimmu.2026.1843872

**Published:** 2026-05-21

**Authors:** Shuang Chen, Hongshan Liu, Kaizhong Wang, Hui Wang, Anqiao Xu, Moran Suo, Xin Chen, Zhonghai Li

**Affiliations:** 1 Department of Orthopedics, First Affiliated Hospital of Dalian Medical University, Dalian, Liaoning, China; 2 Key Laboratory of Molecular Mechanism for Repair and Remodeling of Orthopedic Diseases, Dalian, Liaoning, China

**Keywords:** adipokines, autophagy, endoplasmic reticulum stress, ferroptosis, intervertebral disc degeneration, lipid metabolism disorder, oxidative stress

## Abstract

With the global population aging, intervertebral disc degeneration (IDD), an aging-related disease, has been confirmed to be an important cause of low back pain (LBP). LBP can affect people of all ages and has now become one of the leading noncommunicable diseases, resulting in reduced life expectancy and functional disability in patients. Previous studies have mostly focused on the effects of factors such as mechanical load and genetics on intervertebral disc degeneration. With the deepening of research, the focus has gradually shifted to metabolic disorders, among which lipid metabolism plays a non-negligible role. Lipid metabolism is involved in the construction of cell structure and microenvironment, which is crucial for maintaining normal physiological functions. Abnormal levels of free fatty acids, triglycerides, and cholesterol are widely recognized as risk factors for intervertebral disc degeneration. Lipid metabolism disorders participate in the pathological process of intervertebral disc degeneration through the following pathways: affecting the release of pro-inflammatory cytokines, activating oxidative stress, inducing excessive autophagy, and triggering endoplasmic reticulum stress. Meanwhile, these four pathways can synergistically exacerbate intervertebral disc degeneration. This paper reviews studies on the association between lipid metabolism disorders and intervertebral disc degeneration, focusing on the underlying pathological mechanisms, so as to provide new insights for further exploring the treatment and intervention strategies of intervertebral disc degeneration, and emphasizes the significance of lipid metabolism in maintaining intervertebral disc health.

## Graphical abstract

**Figure:**
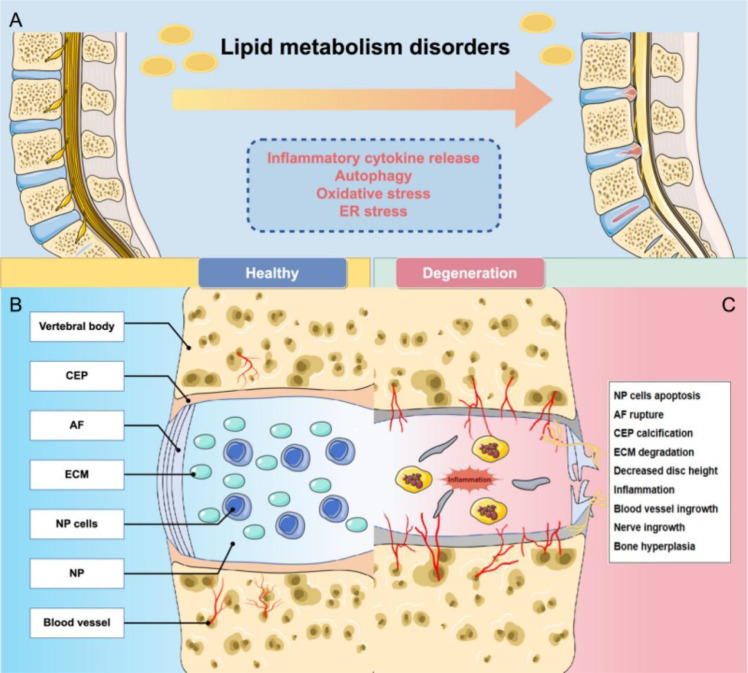
**(A)** Lipid metabolism disorders affect the degenerative changes of the IVD through multiple pathological mechanisms (Inflammatory cytokine release, autophagy, oxidative stress, and ER stress). **(B)** A healthy intervertebral disc is primarily composed of NP, AF, and CEP, serving as the structure that connects two adjacent vertebrae. The ECM is generated and maintained by NP cells. The CEP is penetrated by blood vessels, facilitating nutrient exchange between IVD and the bone marrow. **(C)** The IVD is affected by lipid metabolism disorders and undergoes a series of pathological changes(NP cells apoptosis, AF rupture, CEP calcification, ECM degradation, Decreased disc height, Inflammation, Blood vessel ingrowth, nerve ingrowth, Bone hyperplasia, etc.) leading to IDD. IVD, Intervertebral disk; ER, Endoplasmic reticulum; NP, Nucleus pulposus; AF, Annulus fibrosus; CEP, Cartilaginous endplate; ECM, Extracellular matrix; IDD, Intervertebral disc degeneration.

## 1 Introduction

The intervertebral disc (IVD) is a partially movable joint of fibrocartilaginous tissue that connects two adjacent vertebrae in the spine. Intervertebral disc degeneration (IDD) is a natural aging process in the human body and one of the main causes of chronic low back pain (LBP) (1). Approximately 40% of patients with LBP have degeneration of the IVD (2). LBP is a common disease of the musculoskeletal system, with 70–85% of people suffering from back pain at some point in their lives (3). Considering people of all ages, LBP ranks first among noncommunicable diseases that lead to reduced life expectancy and disability and has brought a heavy economic burden to society and individuals (4, 5). Prolapse of the nucleus pulposus (NP), loss of water from the IVD, rupture of the annulus fibrous (AF), and ossification of the cartilage endplate (CEP) are prominent features of IDD (6). The process of IDD involves a variety of complex pathophysiological mechanisms, among which the main factors are as follows: nutritional supply disorders; the role of cytokines; oxidative stress; aging; apoptosis of NP, AF, and endplate (EP) cells; genetic influences; and biomechanical loads (7, 8).

Lipid metabolism, involving the synthesis and metabolism of biomolecules such as triglycerides (TGs), cholesterol, and lipoproteins, significantly influences the cellular aging process (9, 10). Excess fat accumulation induces a chronic inflammatory response throughout the body, which in turn affects the development of IDD (11). Adipose tissue can affect the function of other organs, such as bones, muscles, liver, and heart, by secreting adipokines (12). Adipokines, including leptin, adiponectin, resistin, granulin precursors, and endolipins, are a class of cytokines secreted by fat cells (12, 13). Leptin and resistin promote inflammatory responses and further IDD; conversely, lipocalin and progranulin (PGRN) have anti-inflammatory effects (11–13). Lipid metabolism significantly contributes to normal physiological activities and the maintenance of health.

When lipid metabolism is disrupted, various organs are affected. Over the past several years, extensive research has been carried out by scientists to investigate how disruptions in lipid metabolism contribute to the promotion of IDD. However, the mechanism by which lipid metabolism disorders contribute to IDD is still not fully understood. There is an association between blood lipid levels and LBP. IDD, as a causative factor of LBP, may be affected by lipid levels (14). One study revealed that lipid metabolism disorders are related to IDD (15). Obesity (OB) is significantly associated with IDD (15). The abnormal weight of obese people leads to excessive mechanical load on the lumbar spine, and chronic low-grade inflammation caused by the severe accumulation of adipose tissue accelerates the process of IDD (16). Obese people often have accompanying hyperlipidemia. Hypertriglyceridemia may cause IDD by mediating disc cell apoptosis and extracellular matrix (ECM) degradation. Hypertriglyceridemia is a high-risk factor for IDD (17). Hypercholesterolemia also seems to be a potential factor leading to IDD. Hypercholesterolemia can activate endoplasmic reticulum (ER) stress to induce the apoptosis of NP cells, thereby leading to IDD (18). An in-depth comprehension of the pathways through which disruptions in lipid metabolism lead to the development of IDD can potentially pave the way for innovative therapeutic strategies and intervention methods for managing this condition in the future. This understanding could enable researchers to identify novel targets for drug development, design more effective treatment protocols, and ultimately improve the quality of life for individuals affected by IDD.

From previous studies, IDD research has focused on two main directions: biomechanics and genetics. Axial loading, torsion, and microdamage to the fibrous ring are the primary biomechanical mechanisms contributing to IDD. The primary genetic determinants contributing to IDD are predominantly heritable differences in collagen, aggrecan, and matrix-modifying enzymes. In contrast, lipid metabolism disorders are thought to be a downstream consequence of OB increasing the mechanical load of the spine, rather than a direct biological regulator of IVD. This lack of research could be due to many reasons. First, the IVD is the largest avascular tissue in the human body and has long been considered evidence that systemic metabolic disorders are unlikely to affect IVD cells. Furthermore, the strong epidemiological link between OB and LBP, which has long been attributed to increased mechanical load, has confused the specific effects of lipid metabolism with weight. Meanwhile, research on intervertebral disc-specific lipidomics and lipid signaling has only truly emerged in recent years. For example, a recent study (19) pointed out that hypercholesterolemia, independent of body weight, activates ER stress in NP cells, and the oxidized low-density lipoprotein (ox-LDL)/lipoprotein receptor 1 (LOX-1) signaling pathway drives endplate chondrocyte senescence and calcification, with ferroptosis being activated in degenerated IVD. This confirms that lipid metabolism is not a bystander but an under-recognized driver of IDD. The present review is built on this premise.

## 2 IVD structure and degeneration

### 2.1 The structure and function of the IVD

The IVD is a structure that connects two vertebrae in the spine and consists of three parts: the NP, the AF, and the CEP (1, 20). The NP is located inside the IVD. The NP is a water-rich heterogeneous structure composed of inorganic salts, water, collagen fibers, and glycosaminoglycans (21). NP cells primarily synthesize and maintain the ECM, which is vital for tissue structure and function (22). Programmed death of NP cells and metabolic imbalance of the ECM can lead to IDD (23). The AF surrounds the outside of the IVD in a ring shape and is anchored by the EPs above and below (24, 25). There are two types of collagen fibers in IVDs: type 1 and type 2 fibers. The number of type 2 collagen fibers decreases from the inside to the outside, whereas the number of type 1 collagen fibers increases from the inside to the outside. NP collagen fibers account for 20%, and AF collagen fibers account for 70% (25). The AF is composed of multiple layers of concentric collagen fibers, with the outer layer connecting the vertebral bodies and the inner layer wrapping the NP (26). When the AF ruptures, it stimulates the generation of reactive oxygen species (ROS) and exacerbates the oxidative stress of NP cells (27). Oxidative stress induces apoptosis and autophagy in IVD cells (28). The CEP, as the anatomical boundary between the NP and the AF, can protect the vertebral body from mechanical load damage and has the properties of a semipermeable membrane. The blood vessels in the CEP, which allow the exchange of nutrients and fluids between the IVD and bone marrow, gradually decrease in number with age (21).

### 2.2 Pathology of IDD

IVD aging and the degeneration of the nearby spinal cord tissue gradually appear with age and various factors associated with injury. Reduced disc height, decreased hydration due to water loss, weakened pressure-bearing capacity, dysregulated ECM metabolism, and cellular dysfunction are the main pathological features of IDD (20). The group of exocrine molecules that provide structural and physiological support to cells is known as the ECM (29). In addition, apoptosis and autophagy are important manifestations of IVD cell death (30). The Wnt pathway plays a significant role in regulating the growth, development, and aging of IVDs. It affects the degenerative process of the IVD by regulating apoptosis, senescence, and inflammation (31, 32).

The gradual breakdown of the ECM serves as the triggering mechanism for the onset and progression of IDD. The ECM is a complex and sophisticated network structure that integrates structural and nonstructural protein components and plays a crucial role in maintaining the homeostasis of cells and tissues. In normal physiological processes, dynamic changes in the ECM are an important part of tissue repair and regeneration; however, once its balance is disrupted, that is, when an imbalance occurs, it may trigger abnormal tissue remodeling, which poses a potential threat to the health of the body (33).

The synthesis and decomposition of the ECM in NP cells are finely regulated by a variety of cytokines to maintain a dynamic balance, which significantly influences the health of IVDs. ECM degradation is a process that is precisely regulated by multiple factors, such as aggregate proteases, disintegrins, disintegrin and metalloproteinase with thrombospondin motifs, and matrix metalloproteinases (MMPs) (34). In degenerated IVDs, a significant increase in the expression of a series of ECM-degrading enzymes, such as MMP-1, MMP-3, MMP-7, and MMP-9, as well as ADAMTS-1, ADAMTS-4, ADAMTS-5, and ADAMTS-9, was observed (35). Excessive apoptosis and senescence are important driving forces of IDD, which together contribute to the destruction of IVD structure and functional decline (36, 37).

The presence of senescent NP, AF, and CEP cells has been confirmed in degenerated human IVD tissues (38). These senescent cells secrete large amounts of pro-inflammatory cytokines, matrix-degrading enzymes, and chemokines, thereby creating a detrimental microenvironment (39). These cytokines activate multiple signaling pathways in disc cells, leading to suppressed ECM synthesis, upregulation of catabolic factors, and induction of cell apoptosis and senescence. Furthermore, inflammatory mediators stimulate the production of ROS and nitric oxide (NO), further exacerbating oxidative damage. Importantly, lipid metabolism disorders lead to chronic low-grade systemic inflammation, which can be transmitted to the avascular IVD through microvascular infiltration in the AF and CEP (40, 41). Under dyslipidemic conditions, adipose tissue secretes pro-inflammatory adipokines, while anti-inflammatory adipokines are often suppressed. Consequently, the synergistic interactions among lipid dysregulation, senescence, and inflammation drive the progression of IDD.

The accumulation of senescent cells accelerates degenerative changes in the IVD by inducing persistent inflammation and promoting matrix breakdown (42). Proinflammatory cytokines, including interleukin-1β (IL-1β), IL-6, and tumor necrosis factor-α (TNF-α), are involved in the degradation of the ECM and form a vicious cycle (43). One study suggested that IVDs in an inflammatory environment undergo vascular invasion and that proinflammatory cytokines can promote IDD through the vascularization of the IVD (44). In addition, IDD causes nerve fibers to extend erroneously to areas that were originally devoid of nerves, thereby triggering LBP (45). In this process, inflammatory cytokines promote an imbalance in ECM synthesis and degradation and aggravate pain (46). IL-17 plays a significant role in the occurrence of IDD and radicular pain. Jingkun et al. found that IL-17 mediated the inflammatory response to IVD, a process that was achieved through activation of the p38/c-Fos and JNK/c-Jun signaling pathways in NP cells (47). It is worth mentioning that lipoxin A4 (LXA4), as an endogenous lipid mediator, can inhibit the ERK, JNK, and NF-κB/p65 signaling pathways in the spinal cord, reduce the expression of proinflammatory factors (such as TNF-α and IL-1β), and increase the expression of anti-inflammatory factors (such as TGF-β1 and IL-10), thereby protecting the IVD from inflammation and alleviating radicular pain (48).

### 2.3 Crossing the avascular barrier

Any “systemic metabolism-induced IDD” hypothesis faces a central paradox: the IVD is the largest non-vascular structure in the human body. To resolve this paradox, it is necessary to clarify the specific pathways by which circulating lipids, lipoproteins, and adipokine reach IVD cells. In healthy discs, the main pathway is passive diffusion across the CEP. The CEP is a semipermeable membrane-like structure in which microvascular channels connect the vertebral bone marrow and the central NP; small-molecule lipophilic substances such as free fatty acids, oxysterols, and lysophospholipids diffuse across the interface along the concentration gradient (49, 50). With increasing age and progression of hyperlipidemia, CEP shows progressive calcification, decreased vascular channel volume, decreased water content, and decreased permeability (19, 51). This process deprives NPs of nutrient supply on the one hand, and on the other hand, aggregates lipid-derived oxidation products in the CEP cells themselves, where ox-LDL accumulates and binds to LOX-1 receptors, driving endplate chondrocyte senescence and further calcification (19).

Degeneration itself initiates the invasion of new blood vessels into the IVD. When radial clefts appear in the outer layer of AF, microvascular endothelial cells migrate inward along these defects with concomitant ingrowth of sympathetic and sensory nerve fibers (52). VEGF secreted by stressed AF and NP cells, combined with pro-inflammatory cytokines (IL-1β, TNF-α, IL-8), jointly drive the neovascularization process (53). Once microvessels extend into the AF lining and NP, plasma lipoproteins including LDL and ox-LDL can come into direct contact with disc cells that previously lived in an avascular environment, momentarily exposing tissue otherwise protected from systemic dyslipidemia (54). At this point the avascular barrier has been destroyed. Consequently, microvascular changes in the AF and CEP also serve as a critical structural bridge linking systemic lipid metabolism disorders to local pathological events within the degenerated IVD. Understanding this vascular remodeling is essential for elucidating how circulating lipid abnormalities contribute to the progression of IDD, especially in the absence of direct blood supply to the central disc tissue.

## 3 Lipid metabolism disorders

Lipid metabolism involves the synthesis, breakdown, and transportation of lipids (9), including fatty acids (FAs), cholesterol, and adipokines, in the body. Lipid metabolism is important for the construction of the cellular microenvironment. Lipid-related derivatives such as TGs, phospholipids, and diacylglycerols are essential for maintaining normal cellular structure and function (55). Ghandour et al. demonstrated through biochemical integration that p53-centered regulators play an important role in the regulation of phospholipid metabolism (56). Meanwhile, FAs are important substances for human energy metabolism and cell signal transduction. They are divided into essential FAs and nonessential FAs. Essential FAs need to be obtained from food. The synthesis of some FAs depends on the intake of essential FAs, such as eicosapentaenoic acid and docosahexaenoic acid. Some essential FAs, such as arachidonic acid (AA), play a significant role in the inflammatory response. The metabolites of AA play a dual role in the initiation and resolution of inflammation. Prostaglandin E2 promotes the occurrence of inflammation, while LXA4 promotes the termination of inflammation. In summary, AA has anti-inflammatory effects, is an important immunomodulator, and promotes tissue self-repair (57). FAs ingested through the diet are divided into saturated fatty acids (mainly obtained from animal products) and unsaturated fatty acids (mainly obtained from vegetable oils) (58).

FA metabolism is associated with aging, and IDD, as an aging process, is also affected by FA metabolism. Omega-3 FAs possess anti-inflammatory properties. Various n-3 and n-6 FAs, especially AA and LXA4, have anti-inflammatory properties and thus have therapeutic implications for IDD, which is an inflammatory disease (59, 60). Docosahexaenoic acid can activate NF-κB and activator protein-1 to reduce IL-1β-mediated proinflammatory responses in astrocytes (61). saturated fatty acids can activate the toll-like receptor 4 (TLR4) signaling pathway to induce inflammation, thereby promoting aging (61, 62). Thus, the effects of FAs on health are not just related to inflammation, and the specific mechanisms involved are complex.

Approximately 30% of total cholesterol comes from dietary intake, and the remainder comes from the synthesis in organs such as the liver (63). Cholesterol is carried in the bloodstream by low-density lipoprotein (LDL), very-low-density lipoprotein, and high-density lipoprotein (HDL). Cholesterol not only maintains cell membrane fluidity and stability but also plays a crucial role in cell signal transduction. Moreover, cholesterol is involved in the synthesis of bile salts and steroid hormones (64). Cholesterol is a vital component for sustaining optimal body health and supporting essential physiological functions.

Notably, white adipose tissue (WAT) and other mesenchymal cells (such as cartilage and bone) can secrete bioactive molecules, namely, adipokines, which are ways for WAT to participate in the physiological activities of other tissues or organs (65, 66). Adipokines are not only involved in the body’s metabolic processes but are also key players in the immune system and active regulators of inflammatory responses (67). An important way in which disorders of lipid metabolism can have an impact on IDD is via adipokines. Recent studies have shown that adipokines and their receptors, including leptin, adiponectin, resistin, PGRN, visfatin, and lipocalin, are expressed in the IVD. Adipokines affect the pathological process of IDD by exerting proinflammatory or anti-inflammatory effects (11, 13, 68, 69). Lipid metabolism is important for normal life activities, but some diseases occur when lipid metabolism is disrupted. When lipid metabolism is disrupted, FAs and cholesterol accumulate abnormally inside the body, leading to the release of harmful adipokines.

Lipid metabolism disorders have various manifestations, such as OB, hypertriglyceridemia, hypercholesterolemia, and insulin resistance (70). Lipid metabolism disorders trigger senescence and apoptosis in NP, AF, and CEP cells through mechanisms such as causing chronic low-grade inflammation, inducing cellular oxidative stress, mediating ECM degradation, and activating ER stress, which in turn promotes the progression of IDD (17, 18, 41, 69).

## 4 Lipid metabolism disorders influence IDD

Lipid metabolism disorders often cause OB, which represents a manifestation of lipid metabolism disorders. OB is a chronic disease characterized by abnormal or excessive fat accumulation. To define OB more accurately, the concept of body mass index (BMI) was introduced. BMI correlates with the degree of IDD, and weight management is necessary in the prevention of IDD (71). A cross-sectional study by Samartzis et al. revealed that overweight and obese patients had more severe IDD (72). A systematic review by Dario et al. suggested that there is a subtle association between OB and IDD (73). A two-sample Mendelian randomization study by Zhou et al. reported that OB can increase the risk of IDD and LBP (74). In recent years, numerous studies have demonstrated that OB is a significant risk factor for IDD. Being overweight increases the mechanical load on the IVD, which is one of the reasons why OB promotes IDD (75, 76). OB can also affect cellular metabolism by affecting the levels of free FAs (69, 77).

Dyslipidemia contributes to a variety of diseases (such as atherosclerosis), but relatively little research has been conducted on how it affects IDD. A biological experiment in rats revealed that hyperlipidemia can lead to the overexpression of degeneration-related factors in the IVD, thereby accelerating IDD (78). A retrospective study of 790 Chinese patients conducted by Zhang et al. suggested that patients with dyslipidemia have a greater risk of IDD and that blood lipid levels can be a useful indicator for predicting IDD (79). A cross-sectional study by Longo et al. revealed that abnormal blood lipid levels may be risk factors for IDD, but the causal relationship is unclear (77). A retrospective study by Huang et al. involving 302 patients reported that abnormal HDL and TG levels are closely related to the severity of IDD (80). Yuan et al. conducted a cross-sectional study on 1035 Chinese citizens and reported that high total serum cholesterol levels and high LDL levels are independent risk factors for IDD in Chinese people (81). The promotion of IDD by blood lipid levels is achieved through multiple pathways, and further research is needed (**Table 1**).

**Table 1:** Epidemiological and preclinical evidence linking lipid metabolism disorders to IDD.

Year	Authors	Journal	Study category	Outcomes	References
2023	Yuan L et al.	BMC Public Health	Clinical study	High serum TC levels and high LDL levels are independent risk factors for IDD in Chinese people.	(81)
2022	Huang Z et al.	Front Nutr	Clinical study	Abnormal HDL and TG levels are closely related to the severity of IDD	(80)
2021	Zhou J et al.	Front Endocrinol (Lausanne)	Epidemiological study	Two-sample Mendelian randomization (UK Biobank) reported a causal effect of genetically predicted BMI on disc degeneration, sciatica, and LBP, with OR per 1-SD BMI ≈ 1.4.	(74)
2019	Zhang Y et al.	J Orthop Sci	Preclinical animal study	Hyperlipidemia upregulated MMP-3, MMP-13, and ADAMTS-5 in NP tissue and accelerated histological grade of disc degeneration over 12 weeks.	(78)
2016	Zhang Y et al.	Lipids Health Dis	Clinical study	Dyslipidaemia may be associated with a higher risk of developing lumbar disc herniation.	(79)
2015	Dario A et al.	Spine J	Clinical study	An analysis of 32 pairs of twins found a significant association between obesity and LBP, even after accounting for genetic and environmental factors.	(73)
2011	Samartzis D et al.	J Bone Joint Surg Am	Clinical study	A higher BMI was significantly associated with an increase in the severity of disc degeneration.	(72)
2011	Longo U et al.	Eur Spine J	Clinical study	Elevated serum total cholesterol and LDL-C, but not HDL-C, were associated with multilevel lumbar disc degeneration on MRI.	(77)

IDD, Intervertebral disc degeneration; TC, Total cholesterol; LDL, Low-density lipoprotein; HDL, High-density lipoprotein; TG, Triacylglycerol; BMI, Body Mass Index; LBP, Low back pain; OB, obesity.

In summary, lipid metabolism disorders are not merely secondary consequences of obesity-related mechanical overload but may act as independent biological drivers of IDD. Even after improvement in body mass index, factors such as dyslipidemia, hypertriglyceridemia, and hypercholesterolemia remain individually associated with the severity of IDD. That is, lipid abnormalities directly contribute to disc matrix degradation, cellular dysfunction, and inflammatory responses, independent of increased spinal loading. Therefore, lipid metabolism disturbances should be recognized as independent risk factors in the pathogenesis of IDD, warranting further mechanistic investigation and targeted therapeutic strategies.

## 5 Mechanisms through which lipid metabolism disorders mediate IDD

### 5.1 Inflammatory cytokine release

A persistent inflammatory response often occurs in IDD. The levels of inflammatory cytokines such as IL-1β and TNF-α are significantly increased in IVDs with inflammation. The activities of these cytokines affect the progression of IDD (46). In previous studies, OB due to lipid metabolism disorders caused IDD through mechanical factors (82, 83). Over the past few years, relevant studies have noted that OB can cause chronic low-grade inflammation throughout the body (11, 67). There is a biochemical link between OB and IDD that is related to metabolism and inflammation (84). Lipid metabolism and adipokine secretion form an elaborate feedback regulatory system. Excessive fat storage or adipocyte hypertrophy caused by lipid metabolism disorders can trigger inflammatory responses such as cellular hypoxia. Meanwhile, it may induce adipocyte dysfunction or apoptosis, accompanied by the release of numerous cellular contents (85, 86). These changes collectively prompt adipose tissue to secrete substantial amounts of adipokines. OB-related inflammatory IDD is associated with the activation of proinflammatory cytokines and their cascades, among which adipokines are important inflammatory mediators (69, 87).

Leptin is the most studied and well-known adipokine and can be secreted by WAT, skeletal muscle, bone, the intestine, and the brain (88). Leptin acts as a crucial predisposing factor for IVD herniation by accelerating IDD and impairing the structural stability of IVDs. Its core mechanism lies in inducing the high expression of matrix metalloproteinase-1 (MMP-1) through the activation of multiple signaling pathways, thereby degrading the ECM of IVDs (89). Meanwhile, IVD cells can produce leptin and express leptin functional receptors. Leptin is involved in the process of IDD (90). Leptin causes IDD mainly by promoting IVD cell proliferation and cytoskeleton remodeling and enhancing ECM degradation (90–92). There is controversy regarding the impact of inflammatory pathways on the progression of IDD. A study by Segar et al. suggested that leptin has a synergistic effect with proinflammatory factors, including TNF-α, IL-1β, and IL-6, increasing the expression of proinflammatory factors, NO, and MMPs and inducing an inflammatory cascade in IVDs (84).

Adiponectin is encoded by the ADIPOQ gene and can regulate the immune system and the expression of MMPs to affect the degradation of the ECM (93). Adiponectin is widely involved in physiological and pathological processes throughout the body and is related to diseases such as heart disease, diabetes, osteoarthritis, and atherosclerosis (94). Only a few studies have investigated whether there is an association between adiponectin and IDD. It has been reported that plasma adiponectin levels affect the severity of IDD (95). However, the IVD is an ischemic tissue, and the correlation between plasma adiponectin and adiponectin in the IVD is limited. Two receptors for adiponectin (AdipoR1 and AdipoR2) are expressed in the NP and AF cells of the human IVD. Adiponectin has been shown to affect the progression of IDD by regulating the levels of TNF-α (94). A study by Yuan et al. suggested that adiponectin expression is downregulated in the NP cells of degenerated IVDs and that the level of adiponectin is related to IDD. In degenerated IVD tissue, a decrease in adiponectin levels may promote an increase in TNF-α secretion by NP cells, thereby accelerating the pathological process of IVDs (96). Moreover, adiponectin exhibited anti-inflammatory effects by inhibiting IL-1β-induced TNF-α expression, which may have a protective effect on the homeostasis of IVD cells (94).

Resistin is a small-molecule protein that is specifically secreted by adipose tissue, which accelerates IDD by regulating the inflammatory and metabolic pathways of nucleus pulposus cells in the IVD (97). In a study by Steppan et al., serum resistin levels were found to be significantly elevated in an obese mouse model. In addition, resistin is considered to be associated with insulin resistance (98). Moreover, resistin can affect lipid metabolism and blood sugar levels (99). Resistin is an inflammatory cytokine associated with inflammatory diseases and can promote the synthesis and release of proinflammatory cytokines (100). OB-associated resistin can synergize with IL-1β to promote the activation of the proinflammatory cytokine cascade in the IVD, thereby promoting the development of IDD (69). A study by Li et al. suggested that resistin can bind to the toll-like receptor 4, activate the NF-κB and p38-MAPK pathways, and upregulate chemokine ligand 4 expression in NP cells. CCL4 enhances the infiltration of macrophages into the IVD and is associated with inflammatory IDD (101).

Another name for visfatin is nicotinamide phosphoribosyltransferase (NAMPT). It is secreted by visceral fat and is a protein associated with insulin sensitivity, oxidative stress, inflammatory responses, and energy metabolism. It profoundly affects the inflammatory process and is associated with a variety of metabolic diseases (102, 103). NAMPT affects cell metabolism by regulating the level of nicotinamide adenine dinucleotide and the activity of NAD-dependent enzymes, affecting cell metabolism and aging (103). NAMPT can influence IDD by participating in the inflammatory response. Cui showed that the MAPK pathway significantly affects inflammation, which is a key factor in the development of IDD. The expression of NAMPT and IL-6 in epidural fat is lower than that in subcutaneous fat. Visfatin activates the JNK/ERK/p38-MAPK signaling pathway and upregulates the expression of IL-6 in NP cells (68). In addition, the results of Huang Y’s study suggested that TNF-α induces ECM degradation and that this process depends on NAMPT regulating the activity of the NOD-like receptor thermal protein domain associated protein 3 inflammasome through the MAPK and NF-κB signaling pathways (104).

Lipocalin (LCN) proteins are a class of small soluble proteins that are widely present in a variety of organisms. LCNs are water-soluble molecules that assist in the transport of hydrophobic molecules such as FAs and steroid hormones in the body (105). LCN2, also known as neutrophil gelatinase-associated lipocalin, is a relatively recently discovered adipokine that plays a key role in inflammatory responses (106–108). It regulates the progression of IDD by modulating oxidative stress, mediating multicellular crosstalk and driving key pathological processes. Liang et al. proposed that Lcn2 serves as a core initiator of the early stress response in IDD, possesses dual pro-oxidative and anti-oxidative properties (109). What’s more, it is specifically highly expressed in degenerative IVD tissues, thus holding the potential to be a therapeutic target. A previous study revealed that the level of LCN2 expression influences adipocyte function and the production of cytokines by macrophages (110). Fan’s study indicated that exosomes released from M1 macrophages effectively deliver LCN2, which activates the NF-κB signaling pathway and further exacerbates IDD by accelerating the senescence process in NP cells (111). Moreover, nerve growth factor interacts with LCN2, and the upregulation of LCN2 enhances the activity of MMP-9, promoting the aging and IDD of AF cells (112, 113). Unfortunately, we lack a sufficient understanding of the association between LCNs and IDD, and the related mechanisms need further study.

**Figure 1:**
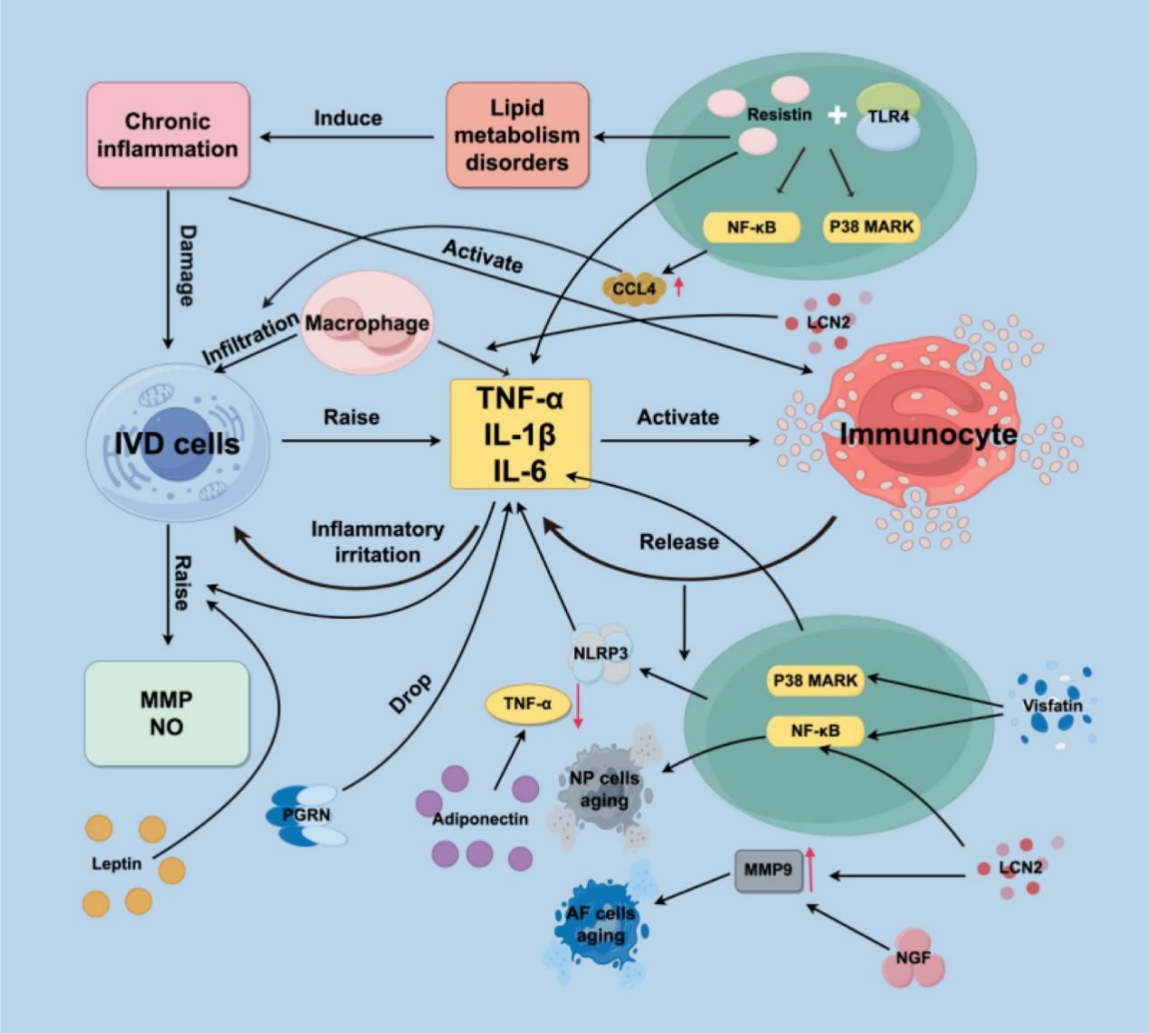
Adipokines affect the release of pro-inflammatory cytokines. IVD, Intervertebral disk; TNF-α, Tumor necrosis factor-α; IL-1β, Interlukin-1β; IL-6, Interlukin-6; MMP, Matrix metalloproteinase; IDD, Intervertebral disc degeneration; TLR4, Toll-like receptor 4; CCL4, chemokine ligand 4; NLRP3, NOD-like receptor thermal protein domain associated protein 3; ECM, Extracellular matrix; LCN2, Lipocalin2; NP, Nucleus pulposus; NGF, Nerve growth factor; AF, Annulus fibrosus; PGRN, Progranulin; IL-10, Interlukin-10; IL-17, Interlukin-17.

PGRN is a growth factor that is secreted by adipose tissue and is rich in cysteine. PGRN is a significant regulatory component of the immune system that may be released by a wide range of cells, including immune cells, epithelial cells, and chondrocytes, among others (114–116). PGRN is involved in both acute and chronic inflammation of the musculoskeletal system and is a regulator of inflammatory responses (117). Research has shown that PGRN plays a role in the inflammatory response triggered by TNF-α. Furthermore, it has the potential to contribute to the process of IDD by influencing the expression of IL-10 and IL-17 (118). A different study suggested that TNF-α plays a significant role in the inflammatory response that occurs in IDD and that atsttrin, which is a derivative of PGRN, has the ability to suppress IDD (119) (**Figure 1**, **Table 2**).

**Table 2:** Adipokines affect the release of proinflammatory cytokines.

Adipokines	Year	Authors	Journal	Outcomes	References
Leptin	2025	Hua, K.F et al.	Int J Mol Sci	Leptin induce the high expression of matrix metalloproteinase-1 through the activation of multiple signaling pathways	(89)
	2019	Segar A et al.	Eur Spine J	Leptin is involved in the process of IDD and has a synergistic effect with pro-inflammatory factors such as TNF-α, IL-1β and IL-6.	(84)
	2015	Miao D et al.	Mol Med Rep	Leptin promotes the catabolism of rat NP cells via MAPK and JAK2/STAT3 pathways.	(92)
	2014	Li Z et al.	Int J Mol Sci	Leptin is a known inflammatory marker and may play a regulatory role in cytoskeletal remodeling in NP cells.	(91)
	2008	Zhao C et al.	Spine (Phila Pa 1976)	IVD cells can express leptin and its functional receptors, and leptin plays a role in the process of IDD.	(90)
Adiponectin	2023	Ohnishi H et al.	Int J Mol Sci	AdipoRon, an agonist that activates the adiponectin receptor, slows IDD by downregulating proinflammatory cytokines.	(93)
	2018	Yuan B et al.	Spine (Phila Pa 1976)	Adiponectin may play an anti-inflammatory role in maintaining IVD homeostasis by downregulating TNF-α production.	(96)
	2016	Terashima Y et al.	J Orthop Surg Res	Adiponectin produced by systemic or epidural adipose tissue may be involved in the pathogenesis of IVD degeneration.	(94)
	2014	Khabour O et al.	Exp Ther Med	There is a strong correlation between plasma adiponectin levels and IDD in Jordanian patients.	(95)
Resistin	2025	Yen, C.K. et al.	JOR Spine	Resistin accelerates intervertebral disc degeneration by regulating the inflammatory and metabolic pathways of nucleus pulposus cells.	(97)
	2017	Li Z et al.	Osteoarthritis Cartilage	Resistin binds to TLR4 and increases CCL4 expression via p38-MAPK and NF-κB signaling pathways in NP cells, which induces macrophage infiltration.	(101)
	2001	Steppan C et al.	Nature	Resistin, a hormone that may link obesity to diabetes, is associated with insulin resistance.	(98)
Visfatin	2021	Cui H et al.	Adipocyte	Visfatin induces IL-6 expression in NP cells via the JNK/ERK/p38-MAPK signaling pathway, thereby promoting IDD.	(68)
	2020	Huang Y et al.	Inflammation	Visfatin downregulation controlled the matrix degradation induced by TNF-α by suppressing NLRP3 inflammasome activity through MAPK and NF-κB signaling in NP cells.	(104)
Lipocalin	2025	Liang, G. et al.	Clin Transl Med	It regulates the progression of IDD by modulating oxidative stress, mediating multicellular crosstalk and driving key pathological processes.	(109)
	2015	Kao T et al.	Eur Spine J	NGF interacts with LCN2, and the upregulation of LCN2 enhances the activity of MMP9, promoting the aging and IDD of AF cells.	(112)
	2014	Kao T et al.	J Neurosurg Spine	NGF may affect the catabolic/anabolic balance of IVD cells and potentiate IDD.	(113)
	2014	Zhang Y et al.	PLoS One	LCN2 is associated with inflammation and adipocyte metabolism, and its secretion and expression are regulated by FA, insulin, glucose, and cytokines.	(108)
	2008	Zhang J et al.	Mol Endocrinol	LCN2 antagonizes the effect of TNF-α on adipocyte inflammation and inhibits the release of cytokines by macrophages.	(110)
Progranulin	2022	Wang C et al.	Neurol Neuroimmunol Neuroinflamm	PGRN is an important regulator of the immune system and plays a protective role in some autoimmune diseases.	(115)
	2018	Wang S et al.	Inflammation	PGRN can inhibit TNF-α-mediated inflammatory response and affect the development of IDD by regulating the expression of IL-10 and IL-17.	(118)
	2017	Ding H et al.	Oncotarget	PGRN exerts anti-inflammatory effects by interfering with TNF-α signaling.	(119)

IDD, Intervertebral disc degeneration; TNF-α, Tumor necrosis factor-α; IL-1β, Interlukin-1β; IL-6,Interlukin-6; NP, Nucleus pulposus; IVD, Intervertebral disc; TLR4, Toll-like receptor 4; CCL4, Chemokine ligand 4; NLRP3, NOD-like receptor thermal protein domain associated protein 3; LCN2, Lipocalin2; AF, Annulus fibrous; NGF, Nerve growth factor; FA, Fatty acids; PGRN, Progranulin; IL-10, Interlukin-10; IL-17, Interlukin-17.

### 5.2 Autophagy

Autophagy is an essential biological process that occurs within cells. During autophagy, damaged or superfluous cellular components, such as proteins and organelles, are carried to lysosomes to be broken down and recycled. This helps to maintain the homeostasis and functioning of the cell (120). Autophagy is related to a wide range of disorders, including cancer, neurodegenerative diseases, and infection, and plays a significant role in a number of physiological and pathological processes. These processes include cell growth and development, as well as the immunological response (121). The target of rapamycin kinase is a key regulator of autophagy (120). There are more than 20 proteins that are closely related to autophagy, which are encoded by evolutionarily conserved autophagy-related genes (122). Mitochondria, peroxisomes, lysosomes, and the ER can all be degraded through autophagy, and lipophagy is a special type of autophagy (123).

In recent years, a significant amount of research has been conducted on the function of autophagy in IDD, and among these studies, there are disputes. In degenerated IVDs, there is a considerable increase in the expression of ATGs, such as Beclin-1, Atg8, Atg12, Cathepsin B, Presenilin 1, and p62 (124). Autophagy is a double-edged sword for IDD. Numerous findings support the idea that autophagy has a protective function in IDD. Autophagy can inhibit ECM degradation, reduce NP cell apoptosis, and improve the CEP inflammatory response and ossification. It has been reported that melatonin can alleviate IDD, increase autophagy, and inhibit the apoptosis and calcification of EP cells under oxidative stress through the Sirt1 signaling pathway (125). The apoptosis and senescence of mouse NP cells are considerably increased, whereas autophagy is suppressed in mouse NP cells that have been treated with bafilomycin A1 (126). However, extensive autophagy activation accelerates IDD (127). A study by Zhan suggested that a long noncoding RNA called HOX transcript antisense intergenic RNA is closely related to autophagy. It can upregulate autophagy to promote programmed cell death in NP cells and ECM degradation (128). Another study revealed that compression can activate autophagy and increase ROS, inducing apoptosis in rat NP cells. In addition, overregulated autophagy triggers autophagic apoptosis (129).

The progression of IDD is induced by factors such as metabolic disorders, mechanical overload, inflammation, and aging. These factors can also lead to mitochondrial dysfunction. Mitochondrial damage is another important pathological basis of IDD (130). There is evidence that mechanical overload can cause mitochondrial dysfunction (131). Both the regular physiological functioning of NP cells and the manufacturing of the ECM are dependent on the normal operation of mitochondria, which is a process in which mitochondrial autophagy plays an important role. BCL2-interacting protein 3 is a mitochondrial outer membrane receptor that regulates mitophagy. Furthermore, mitochondrial dysfunction promotes IDD (132). An excessive amount of mitophagy leads to the death of NP cells, and the HIF1α/NDUFA4L2 pathway is responsible for regulating mitophagy in the presence of oxidative stress (133). Mitochondrial function is impaired in NP cells lacking NLR family member X1, and the PINK1-PRKN pathway is compensatorily activated, inducing excessive mitochondrial autophagy and apoptosis in NP cells (134). Moreover, high glucose can induce oxidative stress, promote mitochondrial dysfunction, and enhance autophagy, thereby promoting the progression of IDD (135). Lipid metabolism disorders lead to an increased mechanical load on IVDs and insulin resistance, but research to clarify the mechanism by which lipid metabolism disorders promote IDD through mitochondrial autophagy is lacking.

Mechanical compression is an important cause of IDD in obese patients with lipid metabolism disorders. The mechanical load has a profound effect on autophagy. Cyclic mechanical tension has been shown to promote IDD. When NP cells are stretched by Cyclic mechanical tension, autophagy increases in the initial stage and then gradually decreases, and ROS levels increase. IDD is accelerated by abnormal mechanical stress, of which ROS and autophagy are regulatory factors (136). Excessive mechanical stress can lead to the upregulation of Piezo1 in IVDs, inhibit autophagy, cause mitochondrial dysfunction, and promote cell apoptosis and ECM degradation (137). Another study reported that compression can regulate NP cell autophagy and upregulate ROS through the JNK/mTOR/AKT/PI3K pathway (138). Importantly, TP53-induced glycolysis and apoptosis regulator is a regulatory factor that controls autophagy and apoptosis in NP cells following mechanical pressure damage. After compression, TP53-induced glycolysis and apoptosis regulator has the ability to prevent apoptosis and autophagy from occurring in NP cells (139).

**Figure 2:**
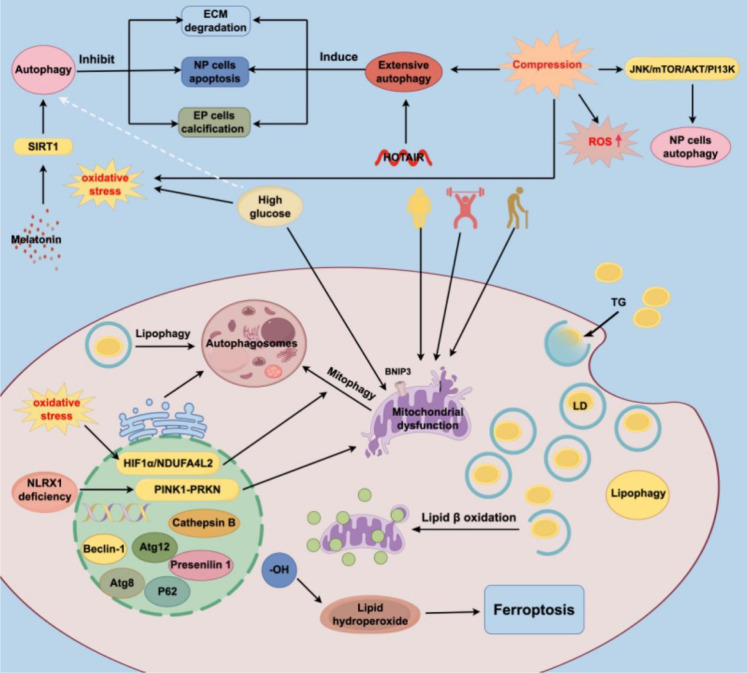
Autophagy is a double-edged sword for IDD. ECM, Extracellular matrix; NP, Nucleus pulposus; EP, endplate; ROS, Reactive oxygen species; HOTAIR, HOX transcript antisense intergenic RNA; IVD, Intervertebral disk; IDD, Intervertebral disc degeneration; BNIP3, BCL2 interacting protein 3; NLRX1, NLR family member X1; LD, Lipid droplets; FA, Fatty acid.

Lipid metabolism can affect the level of lipophagy. In a state of hunger and hypoxia, the body’s nutrition and energy supply require the consumption of lipids, and lipid metabolism and autophagy are linked in this process. Lipid metabolism and storage in cells occur mainly through lipolysis and lipophagy. TG is the most important lipid storage carrier in the body, and its synthesis site is mainly in the liver. TGs usually form lipid droplets and are distributed throughout the body in accordance with the diverse needs of different parts of the body (140). Lipophagy is a distinct autophagic process that specifically targets lipid droplets for degradation as a part of lipid metabolism (141). The release of free FAs from cells depends on lysosomal exocytosis (142). The lipid transfer protein OSBP-related protein 8 is a lipid autophagy receptor on LDs and is essential for lipid metabolism (143). Once the FAs released by LDs are free, they undergo β-oxidation and are degraded by mitochondria to meet the energy needs of the cells (140). Lipid peroxidation is closely related to ferroptosis (144). Lipid autophagy may promote IDD through crosstalk with ferroptosis, but there is a lack of relevant research confirming this notion. Moreover, the inhibition or overactivation of fat autophagy affects the balance of lipid metabolism in the body (145). The activation of lipid autophagy in the context of hyperlipidemia inhibits osteoblast production (146). However, few studies have identified the particular mechanism that causes lipophagy to produce lipid metabolism abnormalities and increase IDD (**Figure 2**; **Table 3**).

**Table 3:** Autophagy is a double-edged sword for IDD.

Year	Authors	Journal	Outcomes	References
2024	Kritschil R et al.	JOR Spine	Autophagy is inhibited in bafilomycin A1-treated mouse NP cells, and the apoptosis and senescence of NP cells are significantly increased.	(126)
2024	Song Y et al.	Autophagy	NLRX1 deficiency can cause mitochondrial dysfunction in NP cells, inducing excessive mitophagy and apoptosis in NP cells.	(134)
2023	Madhu V et al.	Autophagy	BNIP3 regulates mitophagy, and mitochondrial dysfunction promotes IDD.	(132)
2023	Ji C et al.	Bone	Activation of lipophagy in the presence of hyperlipidemia inhibits osteoblast production.	(146)
2022	Shi S et al.	Arthritis Res Ther	Excessive mechanical stress can upregulate Piezo1, inhibit autophagy, cause mitochondrial dysfunction, and promote cell apoptosis and ECM degradation.	(137)
2021	Li Z et al.	Connect Tissue Res	Compression can regulate NP cells autophagy and upregulate ROS through the JNK/mTOR/AKT/PI13K pathway.	(138)
2020	Zhan S et al.	J Cell Physiol	HOTAIR can upregulate autophagy to promote NP cell programmed cell death and ECM degradation.	(128)
2020	Li Z et al.	J Cell Physiol	TIGAR can inhibit the apoptosis and autophagy of NP cells after compression.	(139)
2019	Zhang Z et al.	J Cell Mol Med	Melatonin can alleviate IDD, enhance autophagy, and inhibit apoptosis and calcification of EP cells under oxidative stress through the Sirt1 signaling pathway.	(125)
2019	Xu W et al.	Exp Mol Med	Excessive mitophagy promotes the apoptosis of NP cells, and mitophagy under oxidative stress is regulated by the HIF1α/NDUFA4L2 pathway.	(133)
2019	Yang M et al.	Int J Mol Med	IDD is accelerated by abnormal mechanical stress, and ROS and autophagy are the regulatory factors.	(136)
2013	Ma K et al.	Osteoarthritis Cartilage	Over-regulated autophagy triggers autophagic apoptosis.	(129)
2013	Park E et al.	Int Orthop	High glucose can induce oxidative stress, promote mitochondrial dysfunction, and enhance autophagy, thereby promoting the progression of IDD.	(135)

IDD, Intervertebral disc degeneration; NP, Nucleus pulposus; NLRX1, NLR family member X1; BNIP3, BCL2 interacting protein 3; ECM, Extracellular matrix; ROS, Reactive oxygen species; HOTAIR, HOX transcript antisense intergenic RNA; TIGAR, TP53-induced glycolysis and apoptosis regulator; EP, Endplate.

### 5.3 Oxidative stress

Free radicals that can cause oxidative stress are called ROS and include O_2_^−^, OH^−^, H_2_O_2_, and NO. Free ROS can accumulate abnormally in the body or inside the cell to cause cellular damage, which is a process known as oxidative stress. Harmful stimuli can sometimes lead to an imbalance between antioxidant systems and the free radicals that are present in the cell (147). Some studies have shown that oxidative stress is present in aging IVDs (148). Strojny et al. identified through the detection of key biomarkers that ROS disrupt the stability of the ECM and can induce IVD cell apoptosis, thereby accelerating IDD (149). Many exogenous injuries stimulate disc cells to produce ROS. For example, in response to noxious stimuli, the production of NO and prostaglandin E2 is increased in IVD tissue (150). OB-related disorders of lipid metabolism aggravate the biomechanical loading of the IVD, leading to rupture of the AF (15, 16). Rupture of the AF induces ROS production, causing increased oxidative stress in NP cells (27). A scientific investigation indicated that severe mechanical strain might cause mitochondrial breakdown in NP cells, and the resulting ROS can increase the death of these cells (131).

Disorders of lipid metabolism can also cause chronic low-grade inflammation, and lipid metabolism can induce oxidative stress by causing an increase in ROS through inflammatory pathways (151, 152). ROS in the body originate mostly from cellular immunological responses triggered by inflammatory mediators, including IL-1β, IL-6, IL-17, and TNF-α (46, 153). NLRP3 can induce compromised mitochondrial activity and initiate ROS production in NP cells upon activation by TNF-α (154, 155). ROS themselves are also among the triggering factors of inflammation. ROS increase TNF production, which contributes to ECM breakdown and the release of proinflammatory factors in IVD cells by triggering the NF-κB and MAPK pathways, thereby establishing a positive feedback loop (156, 157). In addition, ROS produced by non-disc cells can induce the production of ROS in disc cells, which is a vicious cycle (158). Notably, ROS levels were significantly increased, and ECM catabolism was significantly increased in AF cells treated with ox-LDL. Moreover, dynamin-related protein-1 (Drp1) expression was increased, and mitochondrial fission was increased in AF cells treated with ox-LDL. In hyperlipidemic conditions, excessive ROS generation and Drp1-mediated mitochondrial fission are associated with ox-LDL-induced AF cell apoptosis (159).

**Figure 3:**
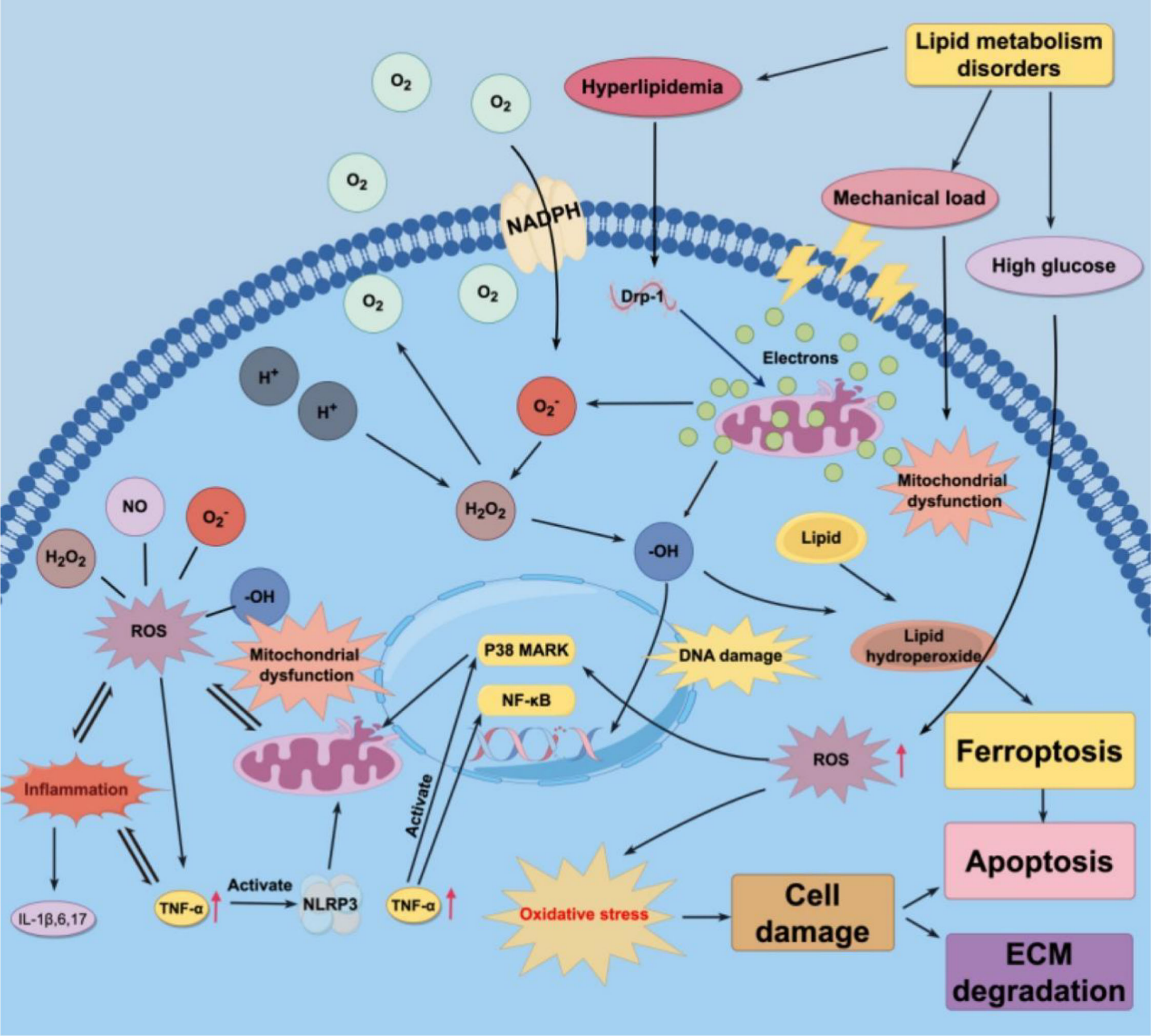
Oxidative stress caused by lipid metabolism disorders promote IDD. ROS, Reactive oxygen species; NADPH, Nicotinamide adenine dinucleotide phosphate; NP, Nucleus pulposus; ECM, Extracellular matrix; NLRP3, NOD-like receptor thermal protein domain associated protein 3; TNF-α, Tumor necrosis factor-α; Drp-1, Dynamin-Related protein-1; AF, Annulus fibrosus; IDD, Intervertebral disc degeneration; DNA, Deoxyribonucleic acid.

Disruptions in lipid metabolism frequently result in insulin resistance, leading to increased blood glucose levels in patients. High glucose stress can cause increased ROS production and activation of the P38 MAPK pathway, leading to mitochondrial dysfunction and the promotion of oxidative stress, which in turn causes NP cell apoptosis and ECM degradation (160). Moreover, respiratory chain defects caused by mitochondrial dysfunction produce a large amount of ROS, and excess ROS can impair mitochondrial function and affect cell viability. Oxidative stress caused by mitochondrial dysfunction is a nonnegligible factor in IDD (161). Oxidative stress also causes lipid peroxidation and DNA damage (147, 155, 162). The level of oxidative stress in the body gradually increases with the accumulation of ROS, leading to ECM degradation and NP cell death or apoptosis in disc tissue, thus causing IDD (163) (**Figure 3**; **Table 4**).

**Table 4:** Oxidative stress and ferroptosis caused by lipid metabolism disorders promote IDD.

Year	Authors	Journal	Outcomes	References
2025	Strojny D., et al.	Front Neurol	ROS disrupt the stability of the extracellular matrix (ECM) and can induce intervertebral disc cell apoptosis, thereby accelerating IVD degeneration.	(149)
2023	Yang X et al.	Adv Sci (Weinh)	Ferroptosis is an oxidative stress-induced cell death pathway characterized by glutathione depletion, and GPX4 is its key regulator.	(164)
2023	Yao B et al.	J Gene Med	The BACH1 promotes IDD by regulating HMOX1/GPX4 to mediate oxidative stress, ferroptosis and lipid metabolism in NP cells.	(171)
2022	Yu H et al.	Oxid Med Cell Longev	The NLRP3 can lead to impaired mitochondrial function and trigger ROS generation in NP cells by being activated by TNF-α.	(155)
2021	Yang R et al.	J Cell Physiol	Ferritin autophagy and oxidative stress can induce ferroptosis and then promote IDD.	(24)
2021	Wu W et al.	J Orthop Res	In the state of hyperlipidemia, excessive ROS generation and Drp1-mediated mitochondrial fission are associated with oxLDL-induced AF cells apoptosis.	(159)
2016	Cheng X et al.	J Diabetes Res	High glucose stress can cause increased ROS and activation of the P38 MARK pathway, which in turn causes NP cell apoptosis and ECM degradation.	(160)
2013	Nasto L et al.	J Orthop Res	There is an association between ROS produced by mitochondrial oxidative metabolism and IDD.	(153)
2013	Gruber H et al.	J Orthop Res	Respiratory chain defects caused by mitochondrial dysfunction produce a large amount of ROS, and excess ROS can impair mitochondrial function and affect cell viability.	(161)
2012	Ding F et al.	Apoptosis	Excessive mechanical load can lead to mitochondrial dysfunction in NP cells, and the ROS generated in this process promotes the apoptosis of NP cells.	(131)
2011	Cheng Y et al.	Biomaterials	The level of oxidative stress in the body gradually increases with the accumulation of ROS, leading to ECM degradation and NP cells death or apoptosis in the disc tissue, thus causing IDD.	(163)
2010	Shamji M et al.	Arthritis Rheum	Rupture of the AF induces ROS production, causing increased oxidative stress in the NP cells.	(27)
2010	Zhou R et al.	Nat Immunol	ROS promotes the degradation of ECM and the secretion of pro-inflammatory factors in IVD cells by promoting the upregulation of TNF expression.	(157)
1997	Kang J et al.	Spine (Phila Pa 1976)	In response to noxious stimuli, the production of NO and PGE2 is increased in IVD tissue.	(150)

IDD, Intervertebral disc degeneration; GPX4, Glutathione peroxidase 4; BACH1, The transcription factor BTB and CNC homology 1; HMOX1, Heme oxygenase 1 gene; NP, Nucleus pulposus; NLRP3, NOD-like receptor thermal protein domain associated protein 3; ROS, Reactive oxygen species; TNF-α, Tumor necrosis factor-α; Drp-1, Dynamin-Related protein-1; oxLDL, Oxidized low-density lipoprotein; AF, Annulus fibrous; ECM, Extracellular matrix; IVD, Intervertebral disc; PGE2, Prostaglandin E2.

### 5.4 Ferroptosis

Oxidative stress can cause the development of IDD by inducing ferroptosis in AF and NP cells. Ferroptosis is a type of cellular death reliant on iron and is distinguished by the peroxidation of phospholipids. Ferroptosis is a cell death mechanism triggered by oxidative stress and marked by glutathione depletion, with glutathione peroxidase 4 (GPX4) serving as its principal regulator (164, 165). Lipoxygenase and P450 oxidoreductase are key metabolic enzymes for phospholipid peroxidation, which rely on iron for catalysis. A strong correlation exists between lipid metabolism and ferroptosis (166). Lipoxygenases and P450 oxidoreductases are key metabolic enzymes for phospholipid peroxidation and are dependent on iron for catalysis (167). Lipid peroxidation is associated with the ferrous ion (Fe^2+^)-mediated Fenton reaction.

Ferroptosis is increasingly recognized as a major contributor to IDD. Mechanistic studies in disc cells have begun to map specific lipid-metabolic inputs onto this axis. Compared with healthy controls, degenerated human NP and AF tissues exhibit reduced GPX4 and solute carrier family 7 member 11 expression, increased acyl-CoA synthetase long chain family member 4 and lysophosphatidylcholine acyltransferase 3 expression, elevated iron and malondialdehyde levels, and depleted glutathione (168, 169). Liu et al (170). found that low-density lipoprotein-induced ferroptosis promotes IDD through the LOX-1/NF-κB/NOX signaling pathway. One study suggested that the transcription factor BTB and CNC homology 1 can promote ferroptosis by inhibiting GPX4. The transcription factor BTB and CNC homology 1 promotes IDD by regulating the heme oxygenase 1 gene/GPX4 to mediate oxidative stress, ferroptosis, and lipid metabolism in NP cells (171). According to another report, oxidative stress promotes the overexpression of nuclear receptor coactivator 4 to transport more ferritin to autophagosomes, which results in the production of more free iron and thus induces iron death. (**Figure 3**). Collectively, these findings indicate that ferritin autophagy and oxidative stress can induce ferroptosis and subsequently promote IDD (24). PDE4B has been reported to upregulate ACSL4 and downregulate GPX4 in NP cells, and pharmacological inhibition of PDE4 attenuates IDD in rodent models (169). Therapeutically, iron chelators, GPX4 stabilizers, and natural antioxidants (such as melatonin) all attenuate disc cell ferroptosis in preclinical studies (164, 172). The convergence of lipid metabolism, iron handling, and redox homeostasis on a single executable cell death program makes ferroptosis a particularly attractive node for lipid-targeted therapy of IDD.

### 5.5 ER stress

The ER is the largest organelle in the cell and is primarily responsible for the synthesis, transport, and folding of proteins; the production of FAs and steroids; the breakdown and use of glucose; and the accumulation of calcium. In addition, it is responsible for the storage of calcium (173). A variety of types of damage can lead to the disruption of ER homeostasis and the slowing of protein folding, which is called ER stress (174). The unfolded protein response can be activated when there is an abnormal buildup of proteins in cells that are either misfolded or incompletely folded (174). Glucose-regulated protein 78 (GRP78), activating transcription factor 4, and X-box binding protein 1 are some of the ER stress regulators whose gene expression increases as a result of the unfolded protein response. However, prolonged ER stress leads to apoptosis through the release of Ca^2+^ and activation of the C/EBP homologous protein (CHOP) pathway (175–177). Kang et al. proposed that ER stress induces ER dysfunction, triggers the cellular apoptotic program and accelerates the progression of IDD (178). It also activates inflammatory signaling pathways such as NF-κB, promotes the release of inflammatory factors, and further disrupts the IVD microenvironment.

**Figure 4:**
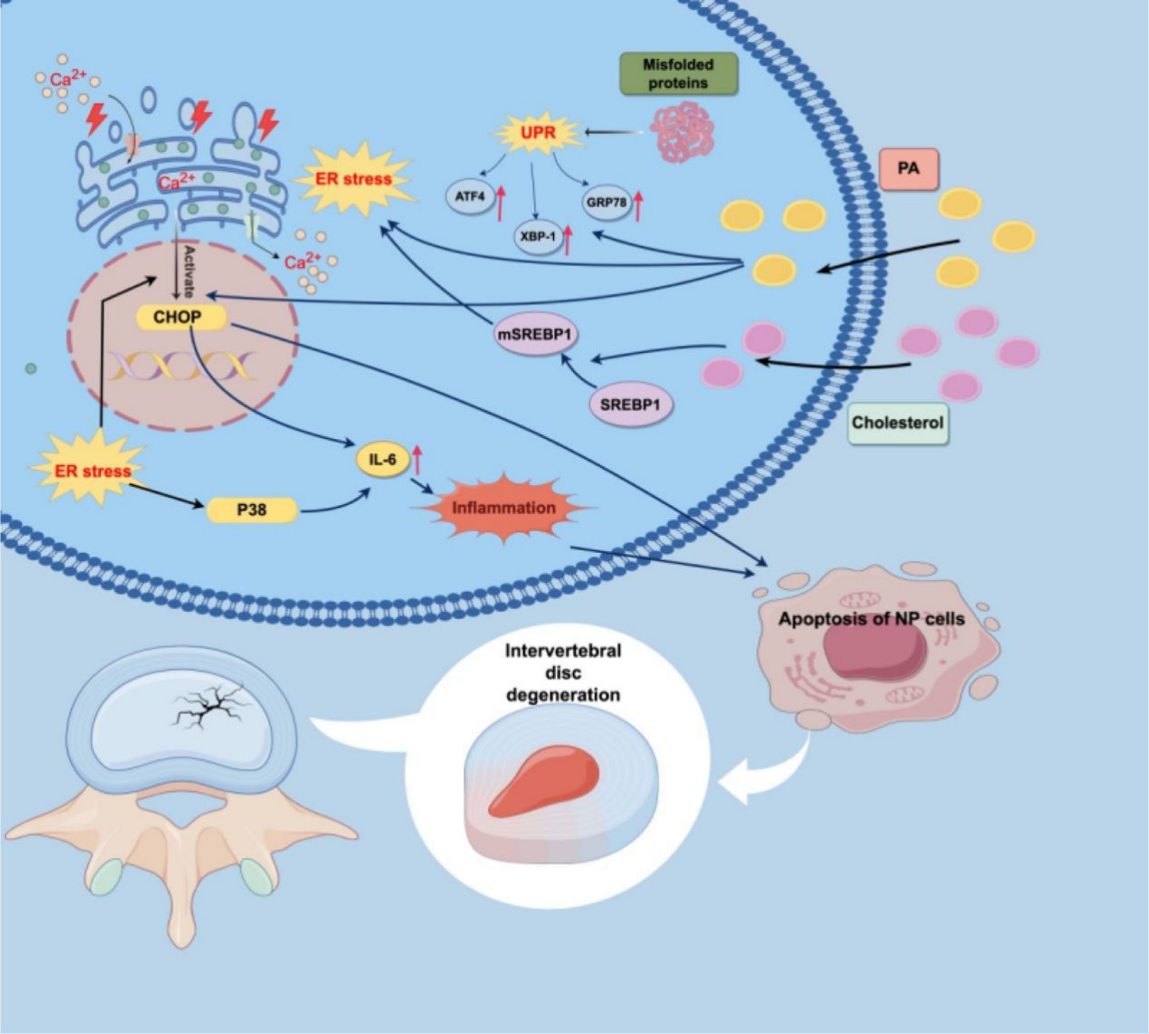
Lipid metabolism disorders cause IDD through ER stress. ER, Endoplasmic reticulum; UPR, Unfolded protein response; GRP78, Glucose-regulated protein 78; ATF4, Activating transcription factors 4; XBP1, X-box binding protein 1; CHOP, C/EBP homologous protein; IL-6, Interlukin-6; IVD, Intervertebral disk; PA, Palmitic acid; NP, Nucleus pulposus; IDD, Intervertebral disc degeneration; SREBP1, Sterol regulatory element-binding protein 1; ECM, Extracellular matrix.

Several studies have reported evidence of ER stress in IDD (179, 180). ER stress can promote the release of IL-6 and induce an inflammatory response in IVDs through the activation of p38 and CHOP. There is crosstalk between ER stress and the release of IL-6 (181). Various metabolites, such as advanced glycation end products and free FAs, can cause IDD through the ER stress pathway (175). palmitic acids are the predominant saturated FAs in the body and can elicit ER stress and dysregulated lipid metabolism in several cell types. These findings indicate that palmitic acids can increase the expression of the GRP78 and CHOP genes in NP cells. palmitic acids can also induce NP cell apoptosis and lipid accumulation through ER stress, ultimately causing IDD (182). Hypercholesterolemia is an aspect of lipid metabolism disorders. Previous studies have shown that excess cholesterol in the blood is closely related to the occurrence of IDD, but the specific mechanism has not yet been fully elucidated (80, 81). A rat scientific experiment indicated that sterol regulatory element-binding protein 1 (SREBP1) and its mature form (mSREBP1) are crucial regulators of the equilibrium of lipids. Cholesterol accumulation can cause IDD through the ER stress pathway. Cholesterol triggers ER stress by facilitating the development of SREBP1, resulting in NP cell death and ECM breakdown (18). The mechanism by which lipid metabolism disorders cause IDD through ER stress needs further study (**Figure 4**; **Table 5**).

**Table 5:** Lipid metabolism disorders cause IDD through ER stress.

Year	Authors	Journal	Outcomes	References
2026	Kang	Adv Sci (Weinh)	ER stress induces endoplasmic reticulum dysfunction and simultaneously activates inflammatory signaling pathways such as NF-κB.	(178)
2024	Chen X et al.	Commun Biol	PA can induce NP cell apoptosis and lipid accumulation through ER stress, ultimately causing IDD.	(182)
2023	Chen Y et al.	Front Pharmacol	Sal003 prevents ER stress in rat NP cells to inhibit apoptosis and ECM degradation.	(179)
2021	Yan J et al.	Front Cell Dev Biol	Cholesterol induces ER stress by promoting the maturation of SREBP1, leading to NP cell apoptosis and ECM degradation.	(18)
2021	Lin H et al.	Oxid Med Cell Longev	Excessive ROS accumulation activates ER stress and causes the accumulation of mitochondrial Ca2+ and induces NP cells apoptosis.	(180)
2019	Luo R et al.	FEBS J	AGEs promote apoptosis through ER stress in human NP cells and exacerbate IDD in rats.	(175)
2018	Krupkova O et al.	Front Immunol	There is crosstalk between ER stress and the release of IL-6.	(181)

IDD, Intervertebral disc degeneration; ER, Endoplasmic reticulum; PA, Palmitic acid; NP, Nucleus pulposus; ECM, Extracellular matrix; SREBP1, Sterol regulatory element-binding protein 1; ROS, Reactive oxygen species; AGEs, Advanced glycation end products; IL-6, Interlukin-6.

### 5.6 Crosstalk among mechanisms

However, the above four pathways do not function independently. In fact, extensive crosstalk exists among these four pathways. PERK is a core kinase of ER stress. Upon activation, PERK not only induces SGs but also reversely regulates oxidative stress levels by modulating the transcription of antioxidant enzymes. STING regulates oxidative stress through non-canonical pathways and directly mediates ER stress. Oxidative stress and ER stress are interconnected and coordinately regulate cellular responses via the STING-PERK-G3BP1 signaling axis (183). The two pathways synergistically amplify cellular stress responses: oxidative stress exacerbates DNA damage, releases more cytoplasmic dsDNA, and enhances ER stress and immune responses. Meanwhile, mild-to-moderate oxidative stress and ER stress induce autophagy activation (184, 185).

ER stress upregulates autophagy-related genes by activating JNK and initiating PERK, thereby serving as a key signal for autophagy activation. In parallel, ROS induces autophagy by activating AMPK and JNK kinases. In turn, autophagy alleviates stress by removing misfolded proteins, restoring ER function, and selectively degrading mitochondria damaged by oxidative stress (186).

ER stress and oxidative stress can also activate inflammatory pathways in adipocytes to upregulate the expression and secretion of many pro-inflammatory adipokines such as leptin. Elevated stress markers impair adipocyte function and inhibit the synthesis and release of anti-inflammatory adipokines such as adiponectin. Meanwhile, leptin typically exacerbates oxidative stress and inflammation while inhibiting protective autophagy (187, 188).

Through the crosstalk among various pathways, synergistic effects including stress response triggering, autophagy system activation, and abnormal adipokine secretion ultimately lead to IVD cell senescence, matrix degradation, inflammatory responses, and structural destruction, resulting in IDD.

## 6 Targeting lipid metabolism for the treatment of intervertebral disc degeneration

Current treatments for IDD mainly focus on symptomatic therapy and surgical intervention. However, symptomatic treatment cannot cure IDD, and surgical treatment carries risks of complications such as invasiveness and infection. At present, drugs that regulate lipid metabolism mainly include peroxisome proliferator-activated receptor modulators, thyroid hormone receptor β agonists, farnesoid X receptor agonists, inhibitors of key enzymes in intrahepatic *de novo* lipogenesis, and others (189–191).

As can be seen from the above mechanisms, lipid metabolism plays an important role in the progression of IDD, and ameliorating lipid metabolism disorders has emerged as an alternative strategy for the treatment of IDD. Qin et al. found in a rat study that sulforaphane can effectively inhibit nucleus pulposus cell senescence and IDD by activating LAMP1-mediated lipophagy (192). Tang et al. verified in a rat model of cervical spondylosis that Guiqi Huoxue Capsule can improve lipid metabolism and thereby alleviate IDD by correcting abnormal fatty acid metabolic profiles and inhibiting pro-inflammatory factors (193). Thus, targeting lipid metabolism shows great potential for the treatment of IDD.

The most widely studied drugs are statins. Statins can inhibit the secretion of MMPs in various cell lines, including macrophages, vascular smooth muscle cells, and osteoblasts, with a low risk of side effects (194, 195), showing great potential for effectively suppressing IDD. Statins can regulate multiple mechanisms of lipid metabolism and several core pathological processes of IDD, thus possessing the advantage of pleiotropic therapeutic effects. In addition, their administration routes are relatively flexible, providing great potential for local therapy.

Liu et al. confirmed in a cross-sectional observational study that the lipid metabolism-targeted therapeutic mechanism of statins is associated with IDD, and that statins can alleviate IDD by targeting lipid lowering to inhibit inflammatory factors (196). Zhang et al. reported that rosuvastatin reduces nucleus pulposus cell apoptosis, inhibits the activity of degradative enzymes such as MMPs, and downregulates the release of pro-inflammatory factors, thereby alleviating IDD (197). Zhang et al. further demonstrated in an experimental study of reversing high-fat diet-induced lumbar IVDD in rats that statins reduce cholesterol levels in nucleus pulposus cells, inhibit ER stress-mediated pyroptosis and ECM degradation, and thus suppress IDD. These drugs act on multiple pathological mechanisms of IVDD and simultaneously target several pathways of lipid metabolism, showing pleiotropic therapeutic advantages with relatively few complications and risks (198).

Nevertheless, many problems remain to be urgently solved for drugs regulating lipid metabolism. Most of these drugs undergo systemic metabolism and lack specific targeted delivery systems for the IVD. Moreover, the physiological barrier of the IVD reduces drug delivery efficiency and affects therapeutic efficacy. Meanwhile, further clinical trials on the prevention or treatment of IDD using such lipid-regulating drugs, as well as deeper investigations into their underlying mechanisms, are needed to promote clinical translation.

## 7 Discussion

Lipid metabolism is crucial for the normal function and operation of cells and tissues in the body. When lipid metabolism is disrupted, it severely impairs the function and structure of IVD tissue, potentially accelerating the progression of IDD. OB, a typical manifestation of lipid metabolism disorders, can directly or indirectly promote IDD by increasing the mechanical load on the spine and inducing systemic chronic inflammation.

Excessive production of ROS induced by abnormal lipid metabolism not only directly damages the DNA and mitochondrial function of IVD cells but also triggers ferroptosis through inducing phospholipid peroxidation, emerging as an important novel pathway for IVD cell death. Lipid abnormalities such as hypercholesterolemia and palmitic acids can activate the ER stress pathway, inducing cell apoptosis by upregulating the expression of molecules like GRP78 and CHOP. Autophagy, on the other hand, exhibits a “double-edged sword” effect: moderate activation maintains cellular homeostasis by clearing damaged organelles, while excessive autophagy caused by lipid metabolism disorders accelerates the programmed death of nucleus pulposus (NP) cells and the degradation of the ECM. These mechanisms form a synergistic network through signaling axes such as STING-PERK-G3BP1 and pathways including JNK/AMPK, collectively amplifying the degenerative damage to IVDs.

Targeting lipid metabolism has emerged as a promising strategy for the treatment of IDD. Drugs such as statins, sulforaphane, and Guiqi Huoxue Capsules have demonstrated significant IVD protective effects in experimental studies by regulating lipid metabolism balance, inhibiting inflammatory pathways, and activating lipophagy. Among them, statins, with their pleiotropic advantages, not only lower blood lipid levels but also directly inhibit the secretion of MMPs and ER stress-mediated pyroptosis, laying a solid foundation for clinical translation. However, challenges such as the physiological barrier of IVDs and insufficient targeted drug delivery efficiency need to be addressed through formulation innovation and optimization of drug delivery technologies.

Future research should focus on deepening the understanding of the synergistic mechanisms between lipid metabolism disorders and metabolic syndromes such as insulin resistance and hypertension, revealing the combined impact of multiple metabolic abnormalities on IDD. Meanwhile, through multi-omics integrated analysis, gene editing, and other technologies, key targets in the lipid metabolism regulatory network should be identified to develop more specific therapeutic drugs. Additionally, clinical translation research should be strengthened to explore the feasibility of using blood lipid indicators as risk predictors for IDD and optimize the local delivery system of lipid-regulating drugs to improve treatment accuracy and safety.

In-depth analysis of lipid metabolism mechanisms is expected to provide a new diagnostic and therapeutic paradigm for IDD, ranging from risk early warning to precise intervention, ultimately improving the quality of life of patients.
